# GINv2.0: a comprehensive topological network integrating molecular interactions from multiple knowledge bases

**DOI:** 10.1038/s41540-024-00330-y

**Published:** 2024-01-13

**Authors:** Xiao Chang, Shen Yan, Yizheng Zhang, Yingchun Zhang, Luyang Li, Zhanyu Gao, Xuefei Lin, Xu Chi

**Affiliations:** 1 Department of Dermatology and Venereal Disease, Xuan Wu Hospital, Beijing, 100053 China; 2 Agricultural Information Institute, Chinese Academy of Agricultural Science, Beijing, 100081 China; 3 CAS Key Laboratory of Genomic and Precision Medicine, Beijing Institute of Genomics, Chinese Academy of Sciences and China National Center for Bioinformation, Beijing, 100101 China; 4 University of Chinese Academy of Sciences, Beijing, 100049 China; 5 Key Laboratory of Plant Molecular Physiology, Institute of Botany, Chinese Academy of Sciences, Beijing, 100093 China

**Keywords:** Biochemical networks, Software, Software

## Abstract

Knowledge bases have been instrumental in advancing biological research, facilitating pathway analysis and data visualization, which are now widely employed in the scientific community. Despite the establishment of several prominent knowledge bases focusing on signaling, metabolic networks, or both, integrating these networks into a unified topological network has proven to be challenging. The intricacy of molecular interactions and the diverse formats employed to store and display them contribute to the complexity of this task. In a prior study, we addressed this challenge by introducing a “meta-pathway” structure that integrated the advantages of the Simple Interaction Format (SIF) while accommodating reaction information. Nevertheless, the earlier Global Integrative Network (GIN) was limited to reliance on KEGG alone. Here, we present GIN version 2.0, which incorporates human molecular interaction data from ten distinct knowledge bases, including KEGG, Reactome, and HumanCyc, among others. We standardized the data structure, gene IDs, and chemical IDs, and conducted a comprehensive analysis of the consistency among the ten knowledge bases before combining all unified interactions into GINv2.0. Utilizing GINv2.0, we investigated the glycolysis process and its regulatory proteins, revealing coordinated regulations on glycolysis and autophagy, particularly under glucose starvation. The expanded scope and enhanced capabilities of GINv2.0 provide a valuable resource for comprehensive systems-level analyses in the field of biological research. GINv2.0 can be accessed at: https://github.com/BIGchix/GINv2.0.

## Introduction

Accumulation of evidence regarding molecular interactions in biological processes has paved the way for the construction of various biological networks, including signaling, Protein-Protein Interaction (PPI), metabolic, and gene regulatory networks, among others. These networks have found various applications, ranging from visualizing omics data^1,2^ to enriching gene sets using topology^3^, identifying functional modules^4^, conducting causal analyses^5,6^, and developing computational models to understand the effects of network perturbations on cellular states^7^. Moreover, recent efforts have been directed towards associating changes in biological networks with diseases, leading to the emergence of “disease maps“^8–11^. Undoubtedly, the comprehensiveness and accuracy of biological networks form the fundamental keys for their successful application in network-based research.

A number of popular knowledge bases, such as KEGG^12,13^, Reactome^14^, and BioCyc^15^, hold valuable information on molecular interactions in biological processes. To represent the complex relationships between biological molecules, several languages have been developed, such as KGML, BioPAX^16^, GPML^17^, and SBML^18^. However, converting this information into a comprehensive topological network has been a challenging endeavor, especially when dealing with different types of networks, such as signaling and metabolic networks. These networks often utilize distinct definitions for nodes and edges, leading to confusion and potential misinterpretations.

For instance, in signaling networks, an edge starting from node A and ending in node B, i.e., “node A activates node B”, typically implies that A is an enzyme, while B is the substrate and product of a post-transcriptional modification (PTM) reaction, resulting in the retention of the same names for both the substrate and product. In contrast, metabolic networks involve substantial changes in substrates, leading to the generation of products with new names. Therefore, in a metabolic network, an edge starting from A and ending in B, i.e., “node A generates node B”, refers to A being the substrate, and B being the product in this reaction, which significantly differs from the definitions in signaling networks. Without unifying the definitions of the nodes and edges, direct integration of signaling and metabolic networks may introduce confusion and misguidance.

Various tools have been developed to read and parse these languages, with the ability to convert the information into the Simple Interaction Format (SIF)^1,2^. SIF is a semi-structured format, in which each line specifies a source node, a character string describing the type of the edge(s), and one or more target nodes. However, the conversions often work better for signaling networks than for metabolic networks, as multiple substrates in metabolic reactions can lead to the ambiguity of multiple participants. Consequently, information regarding “who participates which reaction” can be lost during the conversion process.

To address these challenges, knowledge bases often visualize networks with edges pointing to edges, such as KEGG, Reactome, and Wikipathways^19^. Although this visualization is user-friendly, it is not suitable to work with common network analysis algorithms and tools. With mounting evidence suggesting the importance of crosstalk between signaling and metabolic networks, there is an urgent need to integrate these networks into a global integrative network, termed “GIN”. Efforts have been made, but mainly focus on the visualization^20^ or leveraging information from PPI network^21^, leaving the signaling and metabolic networks topologically disconnected.

In this context, we propose a visualization layout called “meta-pathway” to fundamentally unify the topological structure of signaling and metabolic networks. To convert conventional pathways into meta-pathways, we introduce an intermediate node for each reaction in the pathways to represent a conceptual “intermediate” state of molecules in biochemical reactions. In most of biochemical reactions with multiple substrates or at least one enzyme, the substrate(s) and the enzyme need to get close enough to each other for the reactions to proceed, which forms the intermediate state. This intermediate state of the molecules is temporary, and will quickly be converted into products. Therefore, the intermediate nodes which come from the intermediate state of the molecules capture the relationships between molecules in real world, and enables both signaling and metabolic reactions to be considered as chemical reactions, facilitating storage in SIF-like format. By converting the pathways into meta-pathways and merging them, we have successfully built GINs for 7077 species based on KEGG^22^.

In addition to KEGG, multiple biological knowledge bases offer valuable molecular interaction data across various aspects. In this study, we have converted molecular interaction data from ten different knowledge bases into the SIF format with intermediate nodes (referred to as SIFI). Subsequently, we conducted a thorough analysis of the consensus among these interactions before integrating the GINs into a single, comprehensive network, namely GIN for human version 2.0 (GINv2.0). Our results demonstrate that this version of GIN is currently one of the most comprehensive human databases of molecular interactions, allowing for straightforward visualization and interpretation of the crosstalk between signaling and metabolic networks, exemplified through a detailed examination of the glycolysis process and the related regulative proteins.

## Results

### Conversion of BioPAX to SIFI

**Fig. 1:**
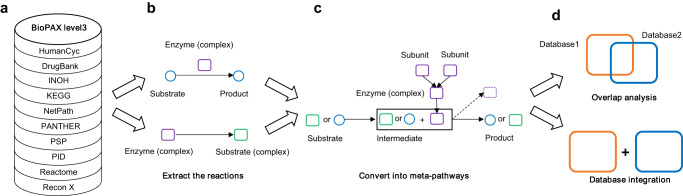
Construction of global integrative network for human. The workflow consists of (**a**, **b**), the extraction of reactions from the BioPAX level3 (owl) files of 10 databases, (**c**) the conversion of the data into meta-pathways, and (**d**) the analysis of overlaps and the integration of the databases. The upper part of (**b**) represents a metabolic reaction and the lower part represents a signaling reaction.

In our efforts to tackle the challenges of different knowledge base languages, we developed a R package named “SIFItools” to efficiently convert BioPAX level 3 owl files from various databases into SIFI format. OWL (Web Ontology Language) format is a powerful and expressive ontology language that allows users to define rich and complex relationships between entities. In the context of Biological Pathway Exchange (BioPAX) language, OWL is used to represent biological pathways and their components, such as molecules, interactions, and cellular processes, in a semantically meaningful way. With SIFItools, we firstly extracted biochemical reactions from the owl files of nine databases prepared by PathwayCommons^23^, including HumanCyc, DrugBank^24^, INOH^25^, KEGG, NetPath^26^, PANTHER^27^, PhosphoSitePlus (PSP)^28^, PID^29^, and Recon X^30^, as well as the owl file of Reactome from its official webpage (not from PathwayCommons). This facilitated the analysis of molecular interactions across multiple databases and laid the groundwork for building a comprehensive network for human cells (Fig. 1a, b). Then each of the reactions was converted into the structure of meta-pathway, introducing an intermediate node (Fig. 1c). After standardization of the 71 ID formats into seven, we analyzed the overlapping genes, chemicals and edges, then integrated all ten databases into one global integrative network, which we refer as GINv2.0 (Fig. 1d).

Notably, although SIFItools automated much of the curation process, manual curation was still necessary due to the diverse naming conventions and special characters used in different databases. In the process of manual curation, the most complicated task involved the conversion of internal IDs from each database’s owl file to corresponding external gene or chemical IDs. This complexity arose from the fact that a single gene or chemical could have different internal IDs across various databases, each linked to one or more distinct external IDs. To overcome this challenge, we developed a two-step approach. Firstly, we constructed an ID mapping table using internal “XRef” links, enabling us to convert the internal IDs to external IDs from 71 different sources. Subsequently, we aggregated the external IDs from diverse sources into gene symbols and unified chemical ID types (UC_IDs), which includes CID^31^, SID^31^, CAS registry number, KEGG, HMDB^32^, and ChEBI^33^ (Supplementary Fig. 1). This method ensured consistency and standardization across the databases, facilitating seamless integration of the data in our subsequent analyses.

### Consensus analysis of the databases

**Fig. 2:**
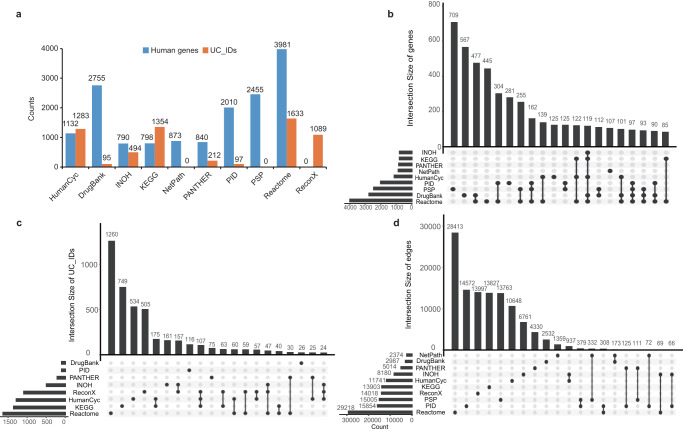
The overlap analysis of the databases. **a** The number of human genes and chemical IDs in the ten databases. **b**–**d** Upset plots showing the overlap of the genes, chemicals and edges between the 10 databases. Each column in the matrix at the lower part of the plot shows the sources of the set whose number is displayed as a bar in the upper part of the plot.

Conversion of BioPAX level3 into SIFI format generated networks varied in the number of edges and nodes, ranging from 873 nodes (NetPath) to 5614 nodes (Reactome) (Fig. 2a), and from 2444 edges (NetPath) to 29898 edges (Reactome) (Supplementary Fig. 2). Notably, the ratio between the quantity of genes and the number of chemicals exhibited variations across the databases. These variations accurately mirrored the distinct scopes of molecular interactions inherent to each individual database. For example, the SIFI format of NetPath and PSP exclusively contained human genes, while Recon X exclusively included chemical IDs (Fig. 2a). This distinction highlights the significance of our integrative approach in capturing a comprehensive picture of human molecular interactions.

Next, we conducted an analysis of the overlapping gene symbols (Fig. 2b), UC_IDs (Fig. 2c), and edges (Fig. 2d) among the ten databases. For clarity, Recon X was excluded from Fig. 2b due to its exclusive focus on chemicals. Similarly, NetPath and PSP were excluded from Fig. 2c. Our analysis revealed that the overlap of gene symbols was notably larger than the overlap of chemical IDs. For instance, in the case of Reactome, the number of unique gene symbols accounted for only 11.2% of its total gene symbols (445 out of 3981), whereas the number of unique chemical IDs represented 76.1% of its total chemical IDs (1243 out of 1633). Furthermore, we found that the overlap of interactions between databases was limited, with over 96.8% (110202 out of 113876) of the interactions being unique to each database for the majority of cases. This observation underscores the distinctiveness and database-specific nature of the interactions. The limited overlap of interactions highlights the importance of our integrative approach in leveraging data from multiple sources to build a comprehensive and interconnected network.

### Integration of the ten databases

**Fig. 3:**
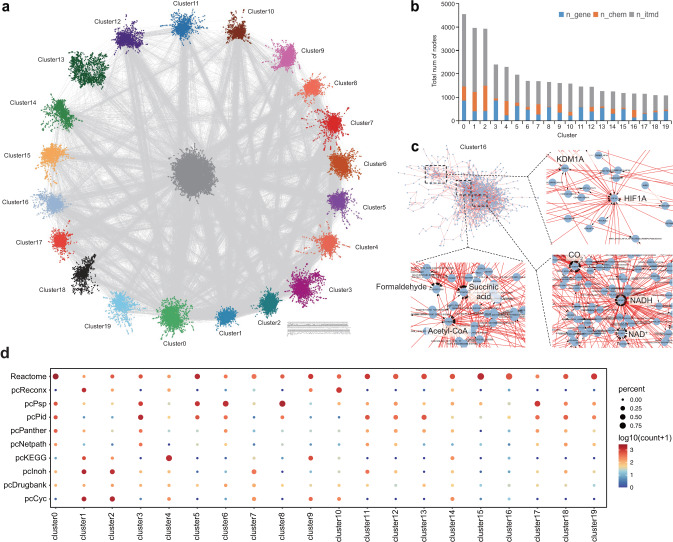
GINv2.0 constructed from 10 databases. **a** Visualization of GINv2.0 by Cytoscape. The largest 20 sub-networks are shown in colors. The small and fragmented interactions which do not connect to the major network are shown on the right bottom. **b** The node composition of the top 20 sub-networks. n_itmd, number of intermediate nodes. **c** Zoomed visualization of cluster16. **d** Dot-plot showing the contribution of different databases to the edges of the clusters. The size of the circle represents the percentage of the edges. The color represents the log10 transformed counts of the overlapping edges.

We merged the SIFI files from all ten databases to construct the raw global integrative network of human. Redundant edges were removed before importing the network into Cytoscape for visualization (Fig. 3a). The final GINv2.0 for human comprises 39,548 nodes and 113,876 edges, encompassing 6330 genes, 3579 chemical IDs, 3957 complexes, and 25,682 intermediate nodes. To facilitate further analysis, we utilized the Python package leidenalg^34^ to cluster the network into distinct sub-networks. In Fig. 3a, we presented the top 20 sub-networks with the largest number of nodes. These sub-networks exhibit diverse compositions of genes, chemicals, and intermediates. Notably, most sub-networks are a mix of genes and chemicals; however, some sub-networks, such as clusters 3, 5, 6, 8, 11, 13, 15, 17, 18, and 19, are predominantly gene-driven, while others, like clusters 4 and 16, are primarily chemical-centric (Fig. 3b). This observation underscores the complex interplay between signaling networks and metabolic pathways, contributing to the complexity of the network.

Additionally, we calculated the topological network metrics, presented in Table 1. Notably, the node with the highest degree was water, followed by ATP and ADP. These findings indicate that water, ATP, and ADP are central participants in biological processes within human cells, aligning well with established knowledge in the field. To gain deeper insights into specific sub-networks, we conducted a focused examination of cluster 16. We identified several nodes with high degrees, including HIF1A, KDM1A, Succinic acid, Acetyl-CoA, Formaldehyde, CO2, NADH, and NAD+ (Fig. 3c).

**Table 1:** Top 20 nodes with highest degrees.

Id	Name	Degree
CID962	Water	3179
CID1038	H+	2925
CID6022	Adenosine-5’-Diphosphate	1933
CID5957	Adenosine-5’-Triphosphate	1498
SID8148096	ATP(4-)	655
CID977	Oxygen	524
CID87642	Coenzyme A	499
CID1004	Phosphoric Acid	490
CID5884	NADPH	486
SID85646635	ADP(3-)	397
CID1061	Phosphate Ion	383
*SRC*	*SRC Proto-Oncogene, Non-Receptor Tyrosine Kinase*	**379**
SID99319226	NAD(1-)	345
SID111978360	Nucleoside Triphosphate(4-)	344
*PRKACA*	*Protein Kinase CAMP-Activated Catalytic Subunit Alpha*	**343**
CID923	Sodium Ion	342
CID4995	Diphosphate(2-)	336
CID21604869	beta-NADH	325
*AKT1*	*AKT Serine/Threonine Kinase 1*	**324**
CID280	Carbon Dioxide	299

The IDs in bold are protein kinases.

To investigate the composition of the database sources of each cluster, we calculated the percentage of the edges contributed by different databases to each cluster (Fig. 3d). Our analysis showed that ReconX’s data (only consists of chemicals) mainly presents in cluster 1 and cluster 10. For cluster 1, there are three major sources, ReconX, INOH, and HumanCyc. In cluster 10, the major sources are ReconX, Reactome, and HumanCyc. Similar results can be observed for PSP and NetPath. These evidences suggest that the databases focusing on only genes or chemicals are well mixed with other databases. On the other hand, KEGG and Reactome contribute the majority of edges of cluster 4 and cluster 16 respectively, which are chemical-centric, and cluster 15 which is gene-centric by Reactome. This suggests that these two comprehensive databases, KEGG and Reactome, who both cover signaling and metabolic pathways, may have distinct scopes of signaling and metabolic reactions.

### Regulation of glycolysis by signaling proteins

**Fig. 4:**
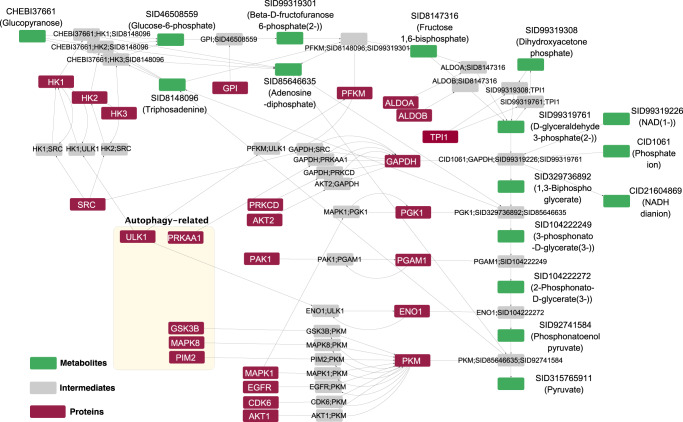
Illustration of glycolysis and its regulative proteins. The regulative protein kinases which are associated with autophagy were clustered in a light-yellow box.

To demonstrate the practical application of GINv2.0 in analyzing signaling and metabolic networks, we extracted nodes representing the metabolites, intermediates, and protein enzymes involved in glycolysis, along with the proteins that regulate these enzymes. Subsequently, we visualized the network in Cytoscape (Fig. 4). Glycolysis is a fundamental cellular metabolic process that converts glucose to pyruvate, generating ATP and NADH. The GINv2.0 visualization of glycolysis clearly illustrates how enzymes are linked to metabolites through intermediate nodes. Moreover, each intermediate node represents a specific reaction, effectively circumventing ambiguity arising from multiple isozymes catalyzing the same reaction.

Subsequently, we focused on the incoming nodes of the enzymes involved in glycolysis, which provided insights into the proteins regulating this crucial metabolic pathway. Our analysis revealed that, out of the ten steps comprising glycolysis, seven steps were regulated by various kinases, including SRC^35,36^, ULK1^37,38^, AKT1^39^, AKT2^40^, PRKAA1^41^, PRKCD^42^, PAK1^43^, MAPK1^44^, MAPK8^45^, GSK3B^46^, PIM2^47^, EGFR^43^, and CDK6^48^. These findings highlight the complicated control mechanisms governing glycolysis, ensuring its harmonious coordination with the activation and inhibition of other cellular pathways, ultimately balancing energy production.

Notably, we found that ULK1 is a prominent positive regulator of HK1^37^, PFKM^37^, and ENO1^37,38^, making it a pivotal protein in governing glycolysis based on the number of controlling enzymes. While SRC is known for its broad involvement in various cellular processes, ULK1 is well known for its essential role in initiating autophagy^49^. Building on this intriguing clue, we deeply explored the relationship between these kinases and autophagy regulation. Remarkably, seven out of the thirteen identified proteins were found to exhibit direct or indirect regulatory effects on autophagy. Protein Kinase AMP-Activated Catalytic Subunit Alpha 1 (PRKAA1), the catalytic subunit of AMPK, plays a crucial role in autophagy initiation under glucose deprivation by directly phosphorylating ULK1^41^. Additionally, AKT suppresses tuberous sclerosis complex proteins 1/2 (TSC1/2) through phosphorylation, leading to mTORC1 activation and subsequent autophagy inhibition^50^. Reports have shown that GSK3B promotes ULK1 acetylation by mediating KAT5/TIP60 phosphorylation during starvation^51^. Furthermore, MAPK8 activates autophagy by mediating BCL2 phosphorylation, facilitating the dissociation of BCL2 from BECN1^52^. Finally, emerging evidence suggests that PIM2 is capable of phosphorylating HK2, thereby promoting autophagy under glucose deprivation^53^.

Collectively, these findings indicate a synergistic regulation of glycolysis and autophagy, particularly under glucose-starved conditions, enriching the understanding of cellular adaptation to varying nutrient availability. In summary, our comprehensive network analysis empowers researchers with fresh perspectives on the cross-talk between metabolic and cellular regulatory networks, paving the way for deeper investigations into the underlying molecular complexities.

## Discussion

In this work, we compiled a much more comprehensive GIN for human compared with the previous version. The previous GIN^22^ for human was built only upon KEGG, which includes 5145 genes and 1501 metabolites. In the present work, we compiled a new GIN for human from ten different databases, which involved 6330 genes and 3579 metabolites, with 23.0% and 138.4% increase, respectively. The new GIN for human is much more useful than the previous one, as the integration of various databases greatly enhances the comprehensiveness of the network. This is exemplified by the demonstration of the orchestrated regulation of autophagy and glucose metabolism under stress, which leveraged information from multiple databases.

We also offer a new tool for the conversion of BioPax level3 files into SIFI format. In our previous work, we built the GIN by a pipeline of perl scripts specifically written to parse KGML files. Since the use of KGML format is currently limited to KEGG, our previous pipeline lacks the ability to process the files of other databases. In our present work, we construct a R package (SIFItools) which can convert BioPax level3 files into SIFI format with minimum manual curations required. Because many biological databases share their data in BioPax level3 format, our new package, SIFItools is more convenient and have much more potential applications when building GIN from databases.

Also in this work, we compared the overlapping information between the 10 databases. We were not able to conduct such analysis in our previous work since we only converted KEGG database into GIN. In our present work, we compared the molecular interactions of the 10 databases and surprisingly found that the overlaps between the databases were rare. Although this could partly due to the different focus of the databases, there is still a great proportion of unmatched ids, especially for metabolites. This could lead to confusing results when applying over-representation analysis (ORA) of pathways, as pointed out by another work^54^.

The knowledge bases of pathways serve as repositories for capturing molecular interactions in both physiological and pathological contexts. While each database emphasizes distinct molecular interactions, synthesizing the collective insights from various sources can outline the comprehensive scope of these knowledge bases. However, the exploration of consensus among diverse knowledge bases has been limited, in part due to the varying data formats used by each database. Despite PathwayCommons’ efforts to standardize data formats, the inherent features of XML format have posed challenges for direct cross-database comparisons. For example, in BioPAX level3, key information about a given reaction may be dispersed across properties such as “left”, “right”, “product”, “controlled”, “controller”, or “cofactor.” This distribution necessitates the extraction of reaction details from multiple attributes to facilitate comparison, thereby complicating and impeding the efficiency of the process. The introduction of meta-pathways and SIFI format has alleviated this predicament by structuring reaction information into a SIF-like three-column configuration. This transformation enables rapid comparisons between reactions, streamlining the comparative analysis.

We noticed that there are overlaps between the concepts of meta-pathway, SIFI format and GIN. To clarify the definitions of the three concepts: (1) Meta-pathway is the way of displaying pathways using intermediates to connect the substrates and products. (2) The GIN (Global Integrative Network) is a network combining the molecular interactions from all pathways. (3) SIFI (Simple Interaction Format with Intermediates) is a format we use to store the molecular interactions of meta-pathways and GIN. The differences between the three concepts are: meta-pathway is the component of GIN, while they can be both stored in SIFI format.

The consensus analysis of GINs generated from different databases highlighted significant diversity across the databases, particularly concerning the edges and nodes related to metabolites. This observed diversity could potentially rise from variations in the specific focus of each database or disparities in naming conventions. Such variations raise valid concerns regarding the reliability of metabolite enrichment analysis, aligning with findings from a recent investigation into the ORA of pathways leveraging metabolomics data. Notably, the authors of this study revealed significant disparities in ORA results when employing distinct databases, such as KEGG, Reactome and BioCyc^54^, which may partially due to the inconsistency we found in our consensus analysis.

The credibility of the edges is also important for network analysis, since questionable edges will create misleading path when conducting path-related network analysis, as evidenced in our previous work^22^. In the comparative analysis of different databases, repeated edges may be more credible since it has been repeatedly validated by different databases. In fact, one of our original goals to compare the edges from different databases was to score the edges based on the number of repeats. However, with the analysis of the databases, we found that a large number of the non-redundant edges are the results of the variations of the scopes of databases. For example, in Fig. 4, the edges extracted from the PSP database are not found in any other databases, but all of these edges have credible sources of publications. This means that a large proportion of non-redundant edges may be credible. Based on this consideration, we excluded the analysis of the credibility of edges in our current work.

By analyzing GINv2.0, we found that the number of intermediate nodes was substantially larger than the combined count of both genes and metabolites. Since each intermediate node represents a distinct biochemical reaction, the number of genes/metabolites involved in a pathway, which is often used in conventional enrichment analysis such as GO, may not truly reflect the number of reactions associated with the pathway. For instance, consider a scenario where five genes are shared between the input gene set and a pathway gene set. While ORA and GSEA^55,56^ might not distinguish whether these five genes participate in one single reaction or five distinct ones, the possibilities of significant associations between the input and pathway gene sets are distinct, judging by instinct. Thus, the intermediate nodes are likely a hidden layer reside between the genes/metabolites and pathways, which has not been investigated for enrichment analysis. The construction of GINs is therefore, a starting point for building the relations between genes/metabolites, intermediate states, and pathways, and further promote the improvement of gene set/pathway analysis.

The illustration of the glycolysis process and the regulative proteins underscores the benefits of the integration of multiple knowledge bases. Notably, we found that the core nodes and edges of the glycolysis process was primarily derived from KEGG, Reactome, HumanCyc, and INOH, while the regulatory interplays between kinases and glycolytic enzymes were from PSP. Individual GINs of any single databases were not able to provide such comprehensive view of molecular interactions. This demonstrates the necessity of database integration to forge a comprehensive and unified network.

In the current version of GIN (v2.0), the intermediate nodes are built for metabolic reactions and PTM reactions, but not for PPI. The reason for excluding PPI is that the GIN we built is a directed graph, but PPI networks are undirected, therefore, current PPI data does not fit for GIN we built. However, we are working on the solution to generate appropriate intermediate nodes for the complexes with multiple protein participants in PPI. With the flexibility of the meta-pathway’s structure, other types of data regarding molecular interactions in cells, including the relations of transcription factors (TFs) and their targets, miRNAs and their targets, will soon be incorporated in GINs as well.

## Methods

### Construction of the Global Integrative Network from ten databases

The owl files of BioPAX level 3 prepared by PathwayCommons were downloaded from https://www.pathwaycommons.org/archives/PC2/v12/. Specifically, we selected DrugBank, HumanCyc, INOH, KEGG, NetPath, PANTHER, PID, PhosphoSitePlus (PSP), and Recon X from PathwayCommons, which contain sufficient number of biochemical reactions extracted from the owl files. The BioPAX level 3 owl file of Reactome was acquired through https://reactome.org/download-data. The owl files were parsed by function “readBiopax” from R package rBiopaxParser^57^, which generated a dataframe for each of the databases.

We built a R package to extract the reactions from the dataframe and convert them into SIFI format. Specifically, we first extracted the reactions from classes of TransportWithBiochemicalReaction, Transport, BiochemicalReaction, ComplexAssembly, Degradation, and Conversion. Then we extracted the information of the enzymes from the classes of Catalysis, Control, and Modulation, and linked the enzymes with the reactions. These information was finally organized into one temporary table.

Subsequently, we created a component matching table designed to capture the relationships between proteins and complexes. We did not use the conventional name of the complexes; instead, we adopted a distinct approach wherein the complexes were systematically deconstructed into constituent proteins through recursive processes. Then the name of the complexes were given by concatenation of all the names of the components in alphabetical order, separated by underscores (“_”) .

Next, we replaced the complex IDs in the reaction table with the name generated from the component names, and convert the reactions into SIFI format. The intermediate nodes were introduced during this conversion step. The names of the intermediate nodes were the concatenation of all the substrates and enzymes, separated by semicolon (“;”). Note that the result of this step still used the local ID system for each owl file specifically which cannot be shared with other owl files.

Since the owl file of each database provides mapping relations between local IDs and commonly used (external) IDs, we replaced the local IDs with the external IDs suggested by each database. However, each databases has its own preference on the use of the ID sources, thus we had to uniform the sources of the IDs to ensure that the same gene/chemical got the same ID in different databases. Uniprot IDs were converted to gene symbols by R package biomaRt^58^. For metabolite IDs, We constructed a mapping table using R package metaboliteIDmapping^59^, and used the strategy in Supplementary Fig. 1 to uniform the IDs with a preference of the sources. A tutorial for the conversion of KEGG’s owl file to SIFI format can be found at https://github.com/BIGchix/SIFItools.

Finally, we concatenated all the SIFI files into one single file, and removed the redundant edges. The edges containing gene IDs of other species were removed.

### Network analysis of human GINv2.0

The intersection results of genes, metabolites and edges were visualized by R package UpSetR^60^. The total network and the network of glycolysis were visualized by Cytoscape^1,2^. The community detection was performed by python package leidenalg^34^, to efficiently work with large directed graph using the Leiden algorithm^34^.

### Reporting summary

Further information on research design is available in the Nature Research Reporting Summary linked to this article.

### Supplementary information

Supplementary Figures

Reporting Summary

## Data Availability

We used the BioPAX level3 owl files prepared by PathwayCommons^23^ which can be accessed here (https://www.pathwaycommons.org/archives/PC2/v12/). The BioPAX level 3 owl file of Reactome was acquired through https://reactome.org/download-data. The GINv2.0 generated in this work can be freely accessed from github: https://github.com/BIGchix/GINv2.0.

## Code availability

The R package “SIFItools” can be freely accessed and installed from github: https://github.com/BIGchix/SIFItools.
