# Supplementary material for: GINv2.0: a comprehensive topological network integrating molecular interactions from multiple knowledge bases
Source: NPJ Syst Biol Appl. 2024 Jan 13;10:4. doi: 10.1038/s41540-024-00330-y (PMC10787761; doi:10.1038/s41540-024-00330-y)
Supplement: Supplementary file 1 — Supplementary Figures [file 41540_2024_330_MOESM1_ESM.pdf]

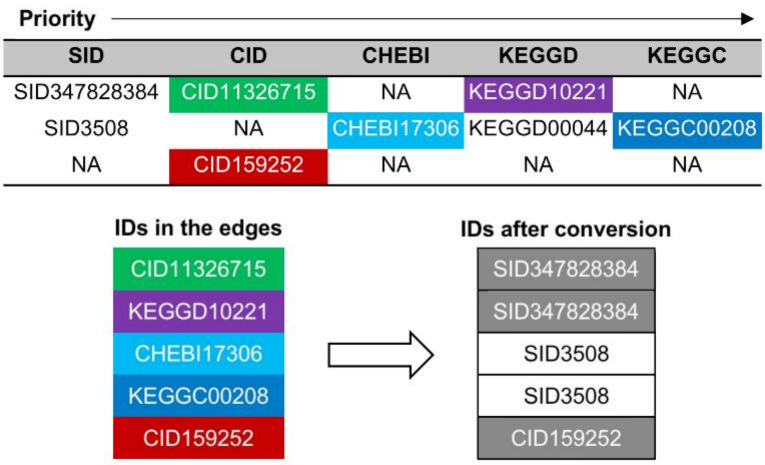

Supplementary Figure 1. The strategy to standardize the ID formats. We first built an ID matching table, then we converted the IDs into preferred ID formats with priority.

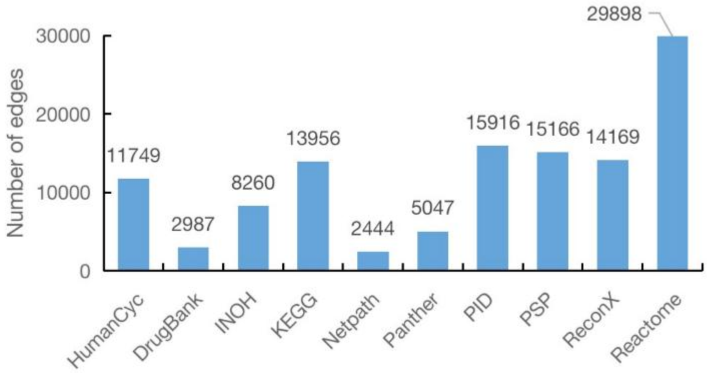

Supplementary Figure 2. The number of edges in the SIFI files generated from the ten databases.
