# Supplementary material for: GINv2.0: a comprehensive topological network integrating molecular interactions from multiple knowledge bases
Source: NPJ Syst Biol Appl. 2024 Jan 13;10:4. doi: 10.1038/s41540-024-00330-y (PMC10787761; doi:10.1038/s41540-024-00330-y)
Supplement: Supplementary file 2 — Reporting Summary [file 41540_2024_330_MOESM2_ESM.pdf]

nature portfolio

Corresponding author(s): Xu Chi
Last updated by author(s): Oct 27, 2023

Reporting Summary

Nature Portfolio wishes to improve the reproducibility of the work that we publish. This form provides structure for consistency and transparency in reporting. For further information on Nature Portfolio policies, see our Editorial Policies and the Editorial Policy Checklist.

Statistics

For all statistical analyses, confirm that the following items are present in the figure legend, table legend, main text, or Methods section.

n/a	Confirmed	
☒	☐	The exact sample size (*n*) for each experimental group/condition, given as a discrete number and unit of measurement
☒	☐	A statement on whether measurements were taken from distinct samples or whether the same sample was measured repeatedly
☒	☐	The statistical test(s) used AND whether they are one- or two-sided<br>*Only common tests should be described solely by name; describe more complex techniques in the Methods section.*
☒	☐	A description of all covariates tested
☒	☐	A description of any assumptions or corrections, such as tests of normality and adjustment for multiple comparisons
☒	☐	A full description of the statistical parameters including central tendency (e.g. means) or other basic estimates (e.g. regression coefficient) AND variation (e.g. standard deviation) or associated estimates of uncertainty (e.g. confidence intervals)
☒	☐	For null hypothesis testing, the test statistic (e.g. *F*, *t*, *r*) with confidence intervals, effect sizes, degrees of freedom and *P* value noted<br>*Give P values as exact values whenever suitable.*
☒	☐	For Bayesian analysis, information on the choice of priors and Markov chain Monte Carlo settings
☒	☐	For hierarchical and complex designs, identification of the appropriate level for tests and full reporting of outcomes
☒	☐	Estimates of effect sizes (e.g. Cohen's *d*, Pearson's *r*), indicating how they were calculated

Our web collection on statistics for biologists contains articles on many of the points above.

Software and code

Policy information about availability of computer code

Data collection	No code was used to collect data in this study.
Data analysis	The R package “SIFItools” built in this work can be freely accessed and installed from github: https://github.com/BIGchix/SIFItools

For manuscripts utilizing custom algorithms or software that are central to the research but not yet described in published literature, software must be made available to editors and reviewers. We strongly encourage code deposition in a community repository (e.g. GitHub). See the Nature Portfolio guidelines for submitting code & software for further information.

Data

Policy information about availability of data

All manuscripts must include a data availability statement. This statement should provide the following information, where applicable:

- Accession codes, unique identifiers, or web links for publicly available datasets
- A description of any restrictions on data availability
- For clinical datasets or third party data, please ensure that the statement adheres to our policy

We used the BioPAX level3 owl files prepared by PathwayCommons23 which can be accessed here (https://www.pathwaycommons.org/archives/PC2/v12/). The BioPAX level 3 owl file of Reactome was acquired through https://reactome.org/download-data. The GINv2.0 generated in this work can be freely accessed from github: https://github.com/BIGchix/GINv2.0

Research involving human participants, their data, or biological material

Policy information about studies with human participants or human data. See also policy information about sex, gender (identity/presentation), and sexual orientation and race, ethnicity and racism.

Reporting on sex and gender	Sex and gender were not considered in this study.
Reporting on race, ethnicity, or other socially relevant groupings	No socially relevant groupings were used in this study.
Population characteristics	There is no human research participant in this study.
Recruitment	There is no human research participant in this study.
Ethics oversight	There is no human research participant in this study.

Note that full information on the approval of the study protocol must also be provided in the manuscript.

Field-specific reporting

Please select the one below that is the best fit for your research. If you are not sure, read the appropriate sections before making your selection.

☒ Life sciences ☐ Behavioural & social sciences ☐ Ecological, evolutionary & environmental sciences

For a reference copy of the document with all sections, see nature.com/documents/nr-reporting-summary-flat.pdf

Life sciences study design

All studies must disclose on these points even when the disclosure is negative.

Sample size	There is no samples used in this study. Our work analyzed the data structure of molecular interaction databases.
Data exclusions	The data of Recon X was excluded from the intersection analysis of genes due to its exclusive focus on chemicals. Similarly, NetPath and PSP were excluded from the intersection analysis of chemicals due to their exclusive focus on genes.
Replication	The result can be replicated following the instructions of our R package "SIFItools".
Randomization	There is no samples used in this study. Our work analyzed the data structure of molecular interaction databases.
Blinding	There is no group allocation issue in this study, because there is no samples used in this study. Our work analyzed the data structure of molecular interaction databases.

Reporting for specific materials, systems and methods

We require information from authors about some types of materials, experimental systems and methods used in many studies. Here, indicate whether each material, system or method listed is relevant to your study. If you are not sure if a list item applies to your research, read the appropriate section before selecting a response.

Materials & experimental systems

n/a	Involved in the study
☒	☐ Antibodies
☒	☐ Eukaryotic cell lines
☒	☐ Palaeontology and archaeology
☒	☐ Animals and other organisms
☒	☐ Clinical data
☒	☐ Dual use research of concern
☒	☐ Plants

Methods

n/a	Involved in the study
☒	☐ ChIP-seq
☒	☐ Flow cytometry
☒	☐ MRI-based neuroimaging

Plants

Seed stocks	This work does not involve plant materials.
Novel plant genotypes	This work does not involve plant genotypes.
Authentication	This work does not involve plant materials nor genotypes.
